# Editorial Notes

**Published:** 1903-12

**Authors:** 


### Editorial Notes.

**University College, Bristol: Faculty of Medicine.**

THE College was delighted to welcome an old and greatly distinguished friend at the annually recurring distribution of prizes to the medical students, performed on October 30th, 1903, by Professor Sir William Ramsay, K.C.B., Ph.D., M.D., F.R.S., who, after distributing the awards, delivered an interesting address on education in general with special reference to the medical student, and the need for increasing specialisation as the sum total of human knowledge increases. It is impossible that any one individual should know everything about everything, but it is equally desirable that most individuals should know everything about something. It is sometimes said that too many are trained to be specialists, and that specialisation has its own evils, but nevertheless the speaker pointed out the impossibility of a complete knowledge of all the subjects of the medical curriculum. He asked what can be done to lessen the load, and taking an illustration from his own subject, Chemistry, he remarked that "it is necessary sadly to confess that the compounds are so complex, the changes so intricate, and the laws of change so obscure, that only a small part of the field has been explored. Is it possible to give a medical student such a training in chemistry that he can make use of discoveries relating to the chemical mechanism of the human body and its functional disorders, so as to be able to form an intelligent opinion as to which suggested remedies should, and which should not, be attempted? I think that the answer must be a decided negative. Even an accomplished organic chemist is far from being in a position to follow the most recent researches in physiological chemistry. The changes which occur in the organism relate not merely to the fact that products are transformed, but to the conditions under which they are transformed—to physical as well as to organic chemistry and the number of men able to handle both divisions of the science is a small one: they are to be found only amongst the most distinguished chemical and physiological investigators of the world. The problems to be solved are almost infinitely complex, and up to the present they have received inadequate study." The remedy suggested by Sir William Ramsay is to divide students into two classes: the 95 per cent. who are to become general practitioners need not be expected to have a profound knowledge of all branches of science up to examination standard, but he of course very rightly holds that every educated man should have some idea of physical science. His proposal is, therefore, "that the first year of a medical student's curriculum should, as now, be devoted to hearing lectures on chemistry, physics, and biology: that he should be required to do the usual class work, including frequent short examinations and exercises to make sure that he understands what he is learning: in a convenient phrase that he should attend the courses to the satisfaction of his teachers, but that he should not be required to show that he has crammed up the subject so as to satisfy an external examiner at the end of his year of study.'

As regards the five per cent. who are to become specialists or investigators, Sir William's view is "that it is impossible to place the standard of knowledge too high. Should we wish to pass regulations on the scope of their education, they might be formulated thus: Such men should first take a science degree, specialising in chemistry; this requires at least three, and better four, years' continuous work; they should be examined at an intermediate stage in chemistry, physics and its necessary mathematics, zoology, and botany, so far as the elements of these sciences are concerned; and, later, they should specialise in organic chemistry, with special attention to physical methods. After, or even before, graduating in science, they should carry out some special research, dealing with these two branches of chemical science. Then, and not till then, should they begin the formal study of a medical career. Their period of incubation, if I may be permitted the expression, should not be less than eight or even nine years. Beginning at the age of 17, at the age of 28 a man may hold a demonstratorship, at 32 a chair. Or, if he choose a specialist's career, at 28 he may assist a specialist; at 35 he may begin to acquire a specialist's practice."

There is an obvious difficulty underlying this scheme, that specialists and investigators are not all made so by heredity or early training: most men hope that they may some day acquire eminence and distinction, and may achieve their aim by the influence of talent, environment and perseverance, rather than by the accidents of early training.

The medical student cannot but be grateful to Sir William Ramsay for his demonstration of the student's burden, and for his endeavour to lighten it.

**Winsley Sanatorium for Consumption.**

We are pleased to observe that the building of the main block, comprising the administration offices, dining-room, etc., and accommodation for twenty patients, is well advanced. The beds are being so rapidly taken up by private donors, various large firms, rural councils, town councils, and local committees, that the Executive Committee have been encouraged—or rather we may truly say have found it necessary—to proceed with the building of a further block to accommodate twenty-two patients more, and this too is now in process of erection. We may remind our readers that the entire use of one bed may be secured by a donation of £250 (which is considerably less than the cost price of building) and an annual subscription of £65 for its maintenance. We fear that when the building is opened some of the public bodies and country towns in the counties of Gloucester, Somerset, and Wilts will find too late that all the available beds have been secured, for a proportion of the beds—certainly not less than one-third—will be reserved for the general use of the subscribers, and therefore will not be allocated. Thus, although the Three Counties' Branch is still sorely in need of further contributions for this excellent and much-needed institution, there are not many beds remaining to be taken up. We observe that the Bristol Corporation has secured altogether twenty beds for our City; and inasmuch as those who thus subscribe for beds will be fully represented on the Executive Committee, it seems a most favourable opportunity for providing for their poorer consumptive citizens at a much smaller cost, both for building and maintenance, than would be possible in a separate institution for twenty patients.

**The King's Sanatorium.** We cannot refrain from referring to the laying of the foundation-stone of this Royal Sanatorium, which is intended to provide for sixty patients. Certain it is that no more beneficent and noble work has ever been conceived and presented to a nation than the Sanatorium so well initiated by His Majesty at Midhurst; yet the King's Sanatorium has not escaped adverse criticism directed against the design, and the expenditure of so large a sum as £100,000 on the buildings. We allude especially to the views advanced by Dr. Reinhardt in favour of the "hut" system, which is far cheaper and would have allowed provision for a much larger number of patients. But it is undeniable that the "single hut" or "chalet" plan has not met with general approval amongst those who have been engaged in the Sanatorium treatment of consumptives, whereas the buildings designed for the King's Sanatorium are the direct outcome of the combined experience of all the better-known and well-tried sanatoria in Germany. That it cannot be taken as a model in cost is obvious; but it is safer and most desirable that it should represent the highest ideals in designs and equipment, so that the results may encourage the erection of many similar, though less costly, sanatoria throughout the kingdom.

**Cheaper Sanatoria** may be built of wood, or "wire-wove," and these have proved in every way as dry, comfortable, and warm as any that are built of more durable material. The cost of building these is about two-fifths the cost of stone buildings.

We note that a new edition of Taylor's *Principles and Practice of Forensic Medicine* is shortly to be published by Messrs. Churchill. We are asked to say that Dr. Fred. J. Smith (138 Harley Street, W.) will be glad to receive accounts of any noteworthy recent medico-legal cases, with dates and references to any trials relating thereto. Dr. Stevenson has placed his collection of poisoning cases at the disposal of the editor, who is particularly anxious to enlarge his collection of other kinds of criminal reports, such as rape, strangulation, etc.

